# Physical Therapists’ Opinion of E-Health Treatment of Chronic Low Back Pain

**DOI:** 10.3390/ijerph18041889

**Published:** 2021-02-16

**Authors:** Jesús Martínez de la Cal, Manuel Fernández-Sánchez, Guillermo Adolfo Matarán-Peñarrocha, Deirdre A. Hurley, Adelaida María Castro-Sánchez, Inmaculada Carmen Lara-Palomo

**Affiliations:** 1 Department of Nursing, Physiotherapy and Medicine, Almeria University, 04120 Almeria, Spain; jesus.martinez@ual.es (J.M.d.l.C.); manuelf@ual.es (M.F.-S.); adelaid@ual.es (A.M.C.-S.); 2 Malaga Health District, Andalusian Health Service, 29009 Málaga, Spain; guillelemur@gmail.com; 3 School of Public Health, Physiotherapy and Sports Science, University College Dublin, Belfield, D04 V1W8 Dublin, Ireland; deirdre.hurleyosing@ucd.ie

**Keywords:** low back pain, physical therapists, home-based exercise, telerehabilitation

## Abstract

(1) Background: Using new technologies to manage home exercise programmes is an approach that allows more patients to benefit from therapy. The objective of this study is to explore physical therapists’ opinions of the efficacy and disadvantages of implementing a web-based telerehabilitation programme for treating chronic low back pain (CLBP). (2) Methods: Nineteen physical therapists from academic and healthcare fields in both the public and private sector participated in the qualitative study. Texts extracted from a transcript of semi-structured, individual, in-depth interviews with each consenting participant were analysed to obtain the participants’ prevailing opinions. The interviews lasted approximately 40 min each. The participants’ responses were recorded. (3) Results: The results suggest that telerehabilitation can only be successful if patients become actively involved in their own treatment. However, exercise programmes for LBP are not always adapted to patient preferences. New technologies allow physical therapists to provide their patients with the follow-up and remote contact they demand, but long-term adherence to treatment stems from knowledge of the exercises and the correct techniques employed by the patients themselves. (4) Conclusions: Physical therapists treating patients with chronic non-specific low back pain believe that new technologies can provide highly effective means of reaching a greater number of patients and achieving significant savings in healthcare costs, despite the limitations of a telerehabilitation approach in developing an appropriate and effective patient-based physiotherapy programme.

## 1. Introduction

Chronic non-specific low back pain (CNLBP) is defined as pain localised in the lumbar spine that persists after initial tissue damage has been treated [1]. Back pain is a recurring problem. Between 42% and 75% of patients still experience LBP after 12 months after inclusion in a study [2]. In the under 45 s, spinal dysfunction is the most common cause of limitations in daily routine activities, while CNLBP is the most common cause of disability in the working population [3]. These symptoms are often the product of a complex mix of biopsychosocial, occupational, and social security factors, which prompt sufferers to seek medical attention. For employers and health insurance companies, treatment for back pain should be geared towards prompt return to work following back pain-related work disability [4]. LBP is one of the most costly musculoskeletal disorders in the world, with a recent study estimating that the direct cost of back pain ranges from 4.2 billion in the Netherlands to 90.6 billion in the United States [5,6].

CNLBP is a complex, poorly understood problem that represents a challenge for different healthcare systems. This is especially evident within the Spanish public health system, where there is a serious problem with waiting lists. Many patients have to wait more than 6 months to receive physical therapy treatment, and physical therapists are forced to treat up to 6 patients within a one-hour timeframe. Exercise rehabilitation is one of the few evidence-based treatments for CNLBP and is recommended in clinical practice guidelines [7]. However, individual success is highly variable and may depend on the patient’s adherence to the recommended exercise programme [8]. Adherence to home exercise programmes ranges from 50% [9] to 70% [10], depending on the studies. Some studies have shown that patients who do not adhere to home exercise regimens benefit less from treatment than those that do [11].

Self-management interventions for chronic pain have been associated with less pain, better functioning, and high levels of patient satisfaction [12,13,14], although some studies have shown that these patients encounter numerous barriers to accessing self-maintenance therapy. These include poor accessibility (for example, there are no services available in many geographic areas and/or they are subject to long waiting times), limited availability of trained professionals (particularly in non-urban centres), and associated treatment costs (e.g., absenteeism) [15,16,17]. Treatment adherence in patients with LBP could be facilitated by using computer-based systems to make exercise programmes more attractive [18]. Irvine et al. [19] explored the efficacy of a mobile app in the self-management of LBP and showed it to be a cost-effective healthcare tool that had the potential to reach a large number of people.

Electronic health interventions (e-Health) promise to help people to cope with chronic diseases. Telerehabilitation (defined for this study as information, computer and communication technology applied to distance rehabilitation programmes between providers and/or patients) is currently considered a promising innovation in solving the problems faced by healthcare systems facing increasing demand due to aging populations, improved treatments and limited resources [20,21,22]. Additionally, some studies have highlighted that e-Health can improve health equity by facilitating access to information and health services [23,24]. However, scientific evidence finds failures in the implementation of such programmes as they do not take into account individual professionals (providers) and the internal and external environment of the patient [25,26,27]. Exploring the contributions and preferences of providers in the design and development process of e-Health programmes is an important step to developing meaningful interventions and possibly strengthening the results of an eHealth self-management intervention for people with chronic low back pain. 

The main aim of this study was to explore physical therapists’ opinions of the efficacy, benefits and disadvantages of implementing a web-based telerehabilitation programme in the treatment of CNLBP. The secondary objective was to explore the experience of physical therapists in the management of patients with CNLBP. 

## 2. Materials and Methods

This study was approved by the Research Ethics Committee of the Department of Nursing, Physiotherapy and Medicine of the University of Almería (EFM 22/19) on 12 December 2019. All participants signed an informed consent form and were fully informed of the objective and characteristics of the study. 

This was a qualitative study based on Gadamer’s hermeneutic approach to data interpretation and analysis. According to Gadamer, a phenomenon can only be understood when a dialogue takes place between the interpreter and the participants through text [28]. In this instance, the text was taken from the transcript of semi-structured, individual, in-depth interviews conducted with each study participant. This technique allowed individuals to express their ideas without interruptions. Consolidated criteria for reporting qualitative research (COREQ checklist) were followed in this study [29].

We used convenience sampling to select physical therapists for the study. The inclusion criteria were that the participants had to be physical therapists with at least 2 years of experience who routinely treat patients with CNLBP. They did not need to have experience in the field of telerehabilitation. The researchers identified a total of 38 physical therapists who met the inclusion criteria. After contacting them by phone (J.M.d.l.C.) to check their availability to participate in the study, five of them rejected the offer because of lack of interest. Sample selection was stopped when data saturation was reached. Finally, a total of 19 physical therapists were included in the study. 

The study was conducted in the province of Almería, Spain, between January and February 2020. All study subjects were asked to sign an informed consent form prior to starting the interviews and collecting socio-demographic data. The in-depth interviews were conducted individually and privately in one of the laboratories of the University of Almería, and each lasted approximately 40 min. Before the interview, participants were thoroughly informed about the web-based exercise programme and the reasons for the investigation. The exercise programme was McKenzie Exercise Therapy and Electroanalgesia based on telerehabilitation, which is a comparable version of rehabilitation performed at home with the help of 10.1 “Quad Core” tablets. Physiotherapists saw the exercise programme that could be assigned to each patient with CLBP, according to the corresponding syndrome (postural, dysfunction and derangement syndrome).

All interviews started with the question, “Could you give your views on the possibility of using a web-based programme for the remote treatment of patients with CNLBP?” and ended with the question “Is there anything else you would like to add on the subject?”. The interviews were conducted by A.M.C.-S., a researcher with 10 years of experience in the treatment of CNLBP and in conducting interviews and managing focus groups to explore emerging issues within the field. None of the participants personally knew the interviewer or their motivations for conducting the research. The participants’ responses were recorded on an audio recorder for subsequent transcription and analysis [30] (only one interview had to be repeated due to a failure in the recording device). Data collection was halted once saturation had been reached. 

The texts were interpreted and codified by the primary researcher (J.M.d.l.C.) and 2 other researchers (A.M.C.-S. and I.C.L.-P.) using thematic analysis techniques guided by the principles of Gadamer’s hermeneutics [28]. In line with the research method, the interview texts were analysed using an adaptation of the stages developed by Fleming [31]. In the first stage, the researchers decided whether the research question was relevant to the methodological assumptions. In the second stage, the researchers identified their prejudices and pre-understandings regarding the treatment of patients with CNLBP. The third stage focused on acquiring understanding through dialogue with study participants, managing to expand on the phenomenon through a fusion of horizons [32]. In the fourth stage, the researchers attempted to gain understanding of the phenomenon through dialogue or an analysis of the text. After rereading the transcripts several times, the participants’ experiences along with the horizons of the researchers were re-examined, and new questions emerged: Is cost saving really necessary? Or would it be better to invest more in human resources? A detailed examination of the transcripts allowed the researchers to inductively identify and extract the themes and sub-themes evident within the transcripts, and further examine their units of meaning. Researchers used the most relevant quotes to support the analysis of results. In the fifth stage, we established the trustworthiness of the extracted qualitative data. To maximise trustworthiness, all data was triangulated between researchers, analysed separately, and any differences were discussed until a consensus was reached. Atlas-ti 7.5 software (Scientific Software Development GmbH, Berlin, Germany) was used to create a single hermeneutic unit where all the transcripts were grouped for subsequent analysis. The transcription and analysis of the data was validated by the participants to ensure their trustworthiness. 

## 3. Results

The final study sample was made up of 19 physical therapists (57.9% men and 42.1% women) with a mean age of 39.36 ± 8.23 years and an age range of 26 and 54 years. The socio-demographic characteristics are shown in Table 1. 

An analysis of the results showed the emergence of 2 main themes that allowed us to understand the physical therapists’ opinion on using an online platform to treat CLBP (Table 2). (1) Patients as active partners in their treatment and (2) new technologies in the treatment of CNLBP.

### 3.1. Patients as Active Partners in Their Treatment

Doctors often take a conservative approach to the treatment of LBP, recommending only rest and pharmacological treatment. There is ample evidence to suggest that exercise is essential for proper recovery and a prompt return to normal activity. It is essential for patients to take an active role in their treatment and take responsibility for their own improvement.

#### 3.1.1. Need for Health Education

Physiotherapists must teach patients how to maintain their health and should correct common misconceptions about LBP. Many patients believe that movement will only aggravate their pain and are therefore extremely apprehensive about any kind of activity (Table 2, theme 1.1.1).

Physiotherapists also need to tailor exercise programmes to the needs of each patient, because not all patients are the same and not all will be able to do the same type of movements or have the same range of movement (Table 2, theme 1.1.2). They must show their patients how to do the exercises correctly and help them perfect the technique to avoid injuring themselves (Table 2, theme 1.1.3).

The patient’s progress must be followed up, and their exercise technique should be corrected from time to time until practitioners are sure they are doing them correctly (Table 2, theme 1.1.4).

#### 3.1.2. Take Responsibility for Their Own Treatment

Physiotherapists in their daily practice observe that their patients contribute little or nothing to resolving their problem. Most patients are used to taking a passive role in which they demand therapy but are unwilling to make any effort themselves. It is essential to make them aware of the need to take responsibility for their own treatment, since maintaining an active lifestyle is essential in this pathology.

Physiotherapists think that it is necessary to change the mentality of the patients and educate them in aspects of health, even amongst the elderly. However, this change in mentality must also affect physiotherapists, who sometimes give up easily (Table 2, theme 1.2.1).

Recent studies show that passive treatment methods like massage therapy or electrotherapy should not be the focus for low back pain. The heavy case load (6 patients per hour) often prevents physiotherapists from spending adequate time with each patient (Table 2, theme 1.2.2).

#### 3.1.3. Emotional Aspect

Physiotherapists observed that patients with CNLBP are usually affected on an emotional level by this disabling condition that eventually impinges on both work and family life. Depression is common, and in many cases the mere fact of being heard and understood improves both their emotional and physical status (Table 2, theme 1.3.1).

When the patient’s pathology becomes chronic, their search for a solution seems hopeless. This can make them feel abandoned or side-lined by medical professionals (Table 2, theme 1.3.2).

Physiotherapists are confident that they can only start to make real progress with the therapy once the patient has understood that their recovery essentially depends on themselves (Table 2, theme 1.3.3).

According to physiotherapists, their greatest challenge lies in getting their patients to believe in the benefits of exercise. It is essential for them to carry on even if their pain persists or even increases at first while doing the exercises (Table 2, theme 1.3.4).

Physiotherapists think that the human contact that occurs in the therapist–patient relationship has an intangible value and cannot be measured. This is perhaps the part that is lost with remote treatment, but that can try to be replaced by other communication channels (Table 2, theme 1.3.5).

### 3.2. New Technologies in the Treatment of CLBP

We live in a world of constant evolution, and what is revolutionary today will be obsolete tomorrow. The same is true of healthcare. Healthcare professionals need to continuously adapt to the latest changes and benefit from all the technological advances available. For the prescription of exercise, there are many technological tools that must be taken advantage of.

#### 3.2.1. Strengths

Physiotherapists have found that patients living in rural, particularly remote, areas do not have easy access to a physiotherapist, and need to be taken by ambulance to the hospital every time they have to receive treatment. This is time-consuming and expensive, and new technologies can lighten the burden (Table 2, theme 2.1.1).

Another major problem in the public health system, in addition to cost, is long waiting lists. Long waiting times usually aggravate a patient’s condition to the extent that it becomes chronic in many cases (Table 2, theme 2.1.2).

Physiotherapists observed that one of the main problems that patients encounter is reconciling family and work life with their therapy. It is important to bear in mind that many patients with CLBP have had to learn to coexist with their pain and go to work normally without taking sick leave. One of the most positive aspects of web-based exercises, in the opinion of physiotherapists, is being able to give such patients a tool that allows them to do their exercises in their own time and receive occasional follow-up online (Table 2, theme 2.1.3).

Another important aspect of therapeutic success is adherence to treatment. Physiotherapists believe that new technologies would make exercising more interesting, particularly for the younger generation. Including a mechanism on the web platform that will enable therapists to check whether they have completed their daily exercise programme can encourage patients to continue with the treatment and adhere to the therapy (Table 2, theme 2.1.4). Introducing a novelty element in the treatment may increase the patient’s expectation of a positive outcome. Some patients with recurrent LBP despite treatment may have lost confidence in the benefit of exercise. Having access to an online platform where they can watch a video that shows them how to do the exercises can change this negative attitude (Table 2, theme 2.1.5).

#### 3.2.2. Weaknesses

According to physiotherapists, the difficulty encountered in using new technologies by older people or those with only basic education could be the greatest obstacle to achieving good results through online therapy (Table 2, theme 2.2.1).

Physiotherapists comment that some people also live in remote, mountainous, or inaccessible regions where internet access can be unreliable (Table 2, theme 2.2.2).

Another generalised theme among study participants was the benefit of direct contact with the patient, allowing them to correct their technique if needed and provide feedback. However, many therapists who work in public hospitals cannot spare the time needed to monitor all the exercises completed by patients (Table 2, theme 2.2.3).

## 4. Discussion

The main aim of our study was to determine and explore physical therapists’ opinions of the effectiveness of a web-based exercise programme for the treatment of CNLBP. After analysing our results, we were able to extract two main themes: “Patients as active partners in their treatment” and “New technologies in the treatment of CLBP”.

Telerehabilitation can only be successful if patients become involved in their own treatment, hence the importance of focusing on patient-centred care. By adopting an approach which focuses on encouraging participation and shared decision-making, active self-management of treatment programmes is facilitated. However, exercise programs for LBP are not always adapted to patient preferences. Patients who have already participated in exercise programs are more likely to participate in programs designed according to their tastes [33]. Qualitative research into patient preferences can help to develop clinical practice and allow therapists to tailor treatments to facilitate patient compliance and satisfaction [34]. Patients are often keen to play an active role in their rehabilitation and resent being ignored [35]. Stenner et al. [36], who explored patient involvement in the decision-making process, concluded that shared decision-making did not appear to be a part of physiotherapy clinical practice. These results should prompt physical therapists to critically appraise their approach when prescribing exercise therapy [37].

Most physiotherapists become frustrated when what they believe is best for their patient does not tally with the patient’s own beliefs and attitudes. In these cases, improving communication skills will help diffuse conflicts [38]. In some studies, the physical therapists themselves suggested that postgraduate training should combine both technical competences and the psychological skills to implement the therapy prescribed [39,40]. Curiously, the participants in our study showed none of the self-criticism that led them to question their own role in the failings they observe.

Perhaps the biggest challenge physical therapists face is therapeutic compliance, which is influenced, among other factors, by the characteristics of the exercise programme and attitude of the therapist. Previous studies show that patients prefer short, simple exercise programmes, and prefer their therapist to be knowledgeable about their disease, encourage feedback, motivate them to learn, give them reminders and monitor their results [41]. New technologies allow physical therapists to provide their patients with the follow-up and remote contact they demand. Such technologies demonstrate the core purpose of e-Health: the utilisation of technological tools at the service of health. Data provided by the relevant literature suggest that such tools provide numerous benefits, including improvement in patient compliance and adherence to treatment [42]. Long-term adherence to treatment stems from knowledge of the exercises and the correct technique to be applied. Study participants agreed that prior practice or one individually supervised face-to-face physiotherapy session in which the patient practices the movements that will be done at home with telerehabilitation tools is the best strategy in ensuring patient adherence [43]. Patients generally abandon their home exercise programmes after a few sessions if they do not get results quickly or when the pain disappears, so we have to try to promote an active lifestyle and self-management to prevent recurrences. Achieving adherence to treatment can be a difficult task and requires continuous patient education [44]. At times, even the best efforts fail, because adherence to the exercises prescribed will depend on the patient’s individual environment, as well as other factors such as personality and historical adherence to other regimens [45].

With respect to the second main theme extracted from our results, new technologies already permeate almost every aspect of our lives. Irvine et al. [19] showed that a mobile-web intervention that tailored content to users’ preferences and interests could be an effective tool in the self-management of LBP. The study supports the notion that mobile apps are cost-effective healthcare tools that can reach a large number of people. Another study, similar to ours in scope but with a focus on patient perspectives of telerehabilitation as opposed to physical therapists, interviewed patients to determine whether they believed that the use of new technologies would improve adherence to exercise programs. It found that expectations differed between older and younger subjects [18], results similar to our own study in terms of how physical therapists think different age groups would respond to such an approach. The previous study observed that younger patients preferred an attractive, challenging interface with the option of recording their performance and modifying their exercise programme, whereas older patients were more interested in receiving guidance and feedback while doing the exercises.

Based on our findings, we consider that an effective method to implement e-Health in clinical care should be divided into 2 phases. First, there should be a face-to-face treatment phase in which a patient-centred clinical practice is adopted, where shared decision-making is encouraged and the importance of physical exercise and the maintenance of an active life promoting self-management is highlighted to the patient. After this, once the patient has assimilated these concepts into their own self-treatment, we propose a second remote phase which allows, through the use of new technologies, the monitoring of the patient’s progress. We also recommend that feedback is given at this stage, thus allowing there to be greater flexibility in the adoption of e-health strategies.

## 5. Strengths and Limitations

The strength of this study lies in the use of qualitative methodology to obtain a clear vision of the different opinions of physical therapists on the possibility of implementing an e-Health programme for the treatment of CNLBP. The trustworthiness of this study was enhanced by a long period of data analysis and independent coding of the data by three members of the research team. This process was fundamental in establishing the emerging themes, sub-themes and units of meaning presented in this study. The use of rigorous methods and the selection of physical therapists from both the public and private fields suggest that our results may be representative of the opinion of a broad range of physical therapists working within a number of different contexts. However, there were some limitations in our study. These included the use of a convenience sample, the limitation of a single, 40-min interview with a participant, which did not allow us to identify possible changes of opinion or shifting perspectives over time, and finally, the impossibility of conducting focus groups for debate which would have further enriched our results.

## 6. Conclusions

New technologies can be a highly effective means of reaching a greater number of patients and achieving significant savings in healthcare costs. The attractiveness and flexibility of web-based exercise programmes promote therapeutic adherence. However, telerehabilitation programmes are only successful when combined with previous psychological work with the patients to raise their awareness of the great importance of self-management and physical activity in their treatment. Furthermore, our studies highlight that for some physical therapists, whilst acknowledging the advantages of an e-Health approach, there may be certain limitations to such a programme. In particular, they can be limiting when defining a correct patient-based physiotherapy programme, or such approaches may be difficult to apply amongst age groups less familiar with new and emerging technologies.

## Figures and Tables

**Table 1:** Selected characteristics of physiotherapists (*n* = 19).

Characteristics	*n* (%)	Mean (SD)	Range
Gender			
Men	11 (57.9)		
Women	8 (42.1)		
Age (years)		39.36 (8.23)	26–54
Professional experience (years)		15.05 (7.38)	4–30
Workplace			
University researcher (UAL)	4 (21.1)		
Private clinic	6 (31.6)		
Public health service (SAS)	9 (47.4)		

UAL: University of Almería; SAS: Andalusian Health Service. SD: standard deviation.

**Table 2:** Themes, subthemes and units of meaning with supporting quotes from the interviews participants (P = 19).

Themes	Subthemes	Units of Meaning	Participant Supporting Quote
1. Patients as active partners in their treatment	1. Need for health education	1. Losing fear of movement	*“I once had a patient in the clinic who had had an episode of low back pain, and for fear of pain had spent a month in bed without moving”* (P3)
		2. Procedural guidelines	*“… we have photocopies of some back exercises and that is what we give all our patients to do at home.”* (P7)
		3. Prior training	*“I always devote the last part of my treatment session to practising the home exercises and correcting their mistakes.”* (P11)
		4. Follow-up and correction	*“There are patients who don’t care how many times they come for treatment; whenever you ask them to do the exercises you have recommended to try to correct them, they continue to do them wrong… That’s because they haven’t done the exercises at home.”* (P1)
	2. Take responsibility for their own treatment	1. Change of mentality	*“Sometimes when I treat elderly patients, I don’t even bother to explain the exercises, it is very difficult for them to understand them and I doubt they would do them anyway…”* (P8)
		2. Passive treatment methods	*“There comes a time when you are so overworked that the easiest thing to do is to connect the device and forget about it…”* (P15)
	3. Emotional aspect	1. Need to feel understood	*“There are times when I think I am more of a psychologist than a physiotherapist… Some patients start to cry, telling me about their problems, and I have not known how to react.”* (P2)
		2. Feeling of abandonment	*“I sometimes try to maintain regular telephone contact with patients who have been given home exercises and who I won’t see again for some time… I think they like you to care about them.”* (P5)
		3. Feeling of self-realisation	*“I had a patient who had never done any kind of physical exercise. At first, she found it very difficult to get into the routine, but as soon as she managed to make it a habit, her pain began to disappear.”* (P19)
		4. Trust in the treatment	*“I get so frustrated when they start to complain as soon as they have started to do the exercise you have sent them…”* (P12)
		5. Human contact	*“I think that simply placing your hand on them and letting them feel cared for is sometimes more important than any therapeutic technique.”* (P3)
2. New technologies in the treatment of chronic low back pain	1. Strengths	1. Cost savings	*“… in the morning shift, 2 or even 3 ambulances arrive every day from outlying villages…”* (P19)
		2. Waiting lists	*“Right now… if it’s not serious… patients have to wait 6 months for their first appointment.”* (P6)
		3. Flexible timetable	*“As soon as they are discharged to return to work, many patients stop going to therapy because it is impossible for them to combine it with their daily life.”* (P1)
		4. Treatment adherence	*“… we are hooked on our mobiles. Now we can be the ones who watch the patients and remind them if they don’t do their home exercises (laughs)…”* (P14)
		5. Outcome expectations	*“… there’s no doubt patients won’t take the same attitude as when you just give them a photocopy of the exercises…”* (P4)
	2. Weaknesses	1. Elderly or poorly educated patients	*“My mother is 76 years old and she doesn’t even know how to send a message on her mobile… I can’t imagine her watching an exercise tutorial on a web page.”* (P11)
		2. Access in rural areas	*“My father is from a small mountain town where there is almost no mobile coverage… and we don’t even talk about the internet (laughs).”* (P15)
		3. Corrections and feedback	*“It would be interesting if patients could periodically come to the clinic to check their progress and correct any mistakes they are making when doing the exercises.”* (P13)

## Data Availability

The data supporting reported results can be obtained through the correspondence author.
